# Orthostasis Is Impaired Due to Fatiguing Intensive Acute Concentric Exercise Succeeded by Isometric Weight-Loaded Wall-Sit in Delayed-Onset Muscle Soreness: A Pilot Study

**DOI:** 10.3390/sports11110209

**Published:** 2023-10-27

**Authors:** Balázs Sonkodi, Tamás Radovits, Emese Csulak, Bence Kopper, Nóra Sydó, Béla Merkely

**Affiliations:** 1 Department of Health Sciences and Sport Medicine, Hungarian University of Sports Science, 1123 Budapest, Hungary; 2 Department of Sports Medicine, Semmelweis University, 1122 Budapest, Hungary; 3 Heart and Vascular Center, Semmelweis University, 1122 Budapest, Hungary; 4 Faculty of Kinesiology, Hungarian University of Sports Science, 1123 Budapest, Hungary

**Keywords:** delayed-onset muscle soreness, Piezo2 channelopathy, Piezo1 ion channel, orthostatic stress test, blood pressure, heart rate, isometric wall-sit

## Abstract

The aim of the study was to investigate any indication of diminished orthostatic tolerance as a result of fatiguing intensive acute concentric exercise with a successive isometric wall-sit followed by an orthostatic stress test, with a special focus on any distinguishable alterations due to a delayed-onset muscle soreness effect. The exercise protocol was carried out among nineteen (10 female, 9 male) junior swimmers from the Hungarian National Swim Team. All athletes showed a positive orthostatic stress test right after our exercise protocol. The diastolic blood pressure was significantly lower due to the delayed-onset muscle soreness effect in the standing position after the supine position of the orthostatic stress test, in contrast to the athletes who did not experience delayed-onset muscle soreness. Furthermore, the heart rate was dysregulated in athletes with a delayed-onset muscle soreness effect when they assumed a supine position after the sustained standing position during the orthostatic stress test, in contrast to the athletes without delayed-onset muscle soreness. Interesting to note is that, in three subjects, the sustained standing position decreased the heart rate below the level of the initial supine position and six athletes experienced dizziness in the standing position, and all of these athletes were from the group that experienced delayed-onset muscle soreness. Accordingly, this study, for the first time, demonstrated that delayed-onset muscle soreness impairs orthostasis after unaccustomed fatiguing intensive acute concentric exercise with a successive isometric weight-loaded wall-sit; however, validation of this association should be investigated in a larger sample size.

## 1. Introduction

Delayed-onset muscle soreness (DOMS) is a late-onset pain condition experienced after unaccustomed and/or strenuous eccentric or isometric exercise but not from concentric exercise [1]. The associated symptoms are as follows: muscle stiffness, swelling, force production loss, reduced range of motion, and impaired proprioception [2]. Several theories have tried to explain this mysterious, largely unknown mechanism for more than 120 years, like lactic acid, muscle spasm, inflammation, connective tissue damage, muscle damage, and enzyme efflux theories [1]. 

One recent neurocentric hypothesis postulates that the primary damage of the bi-phasic injury mechanism of DOMS is a transient Piezo2 channelopathy of the intrafusal proprioceptive terminal [3,4]. According to this argument, this primary microinjury could impair the crosstalk of Piezo1–Piezo2 ion channels; transiently miswire proprioception; activate N-methyl-D-aspartate (NMDA) receptors on the spinal dorsal horn, leading to transient autonomic imbalance; and, last but not least, impair orthostasis [3,4,5,6]. It is widely viewed among scientists that impaired proprioception in DOMS is associated with higher risk of sport injuries, and if DOMS impairs orthostasis, then it could further add to this higher injury risk.

It has been demonstrated that the impairment of baroreceptors could lead to insufficient control of blood pressure due to postural change or physical activity [7,8]. The exact molecular mechanotransductory background of these baroreceptors has not been understood until recently, when it was shown in mice that Piezo2, in conjunction with Piezo1, contributes to the baroreflex [9]. In another study, the same Piezo2 ion channel was found to be primarily responsible for baroreceptor activity [10], as the microinjury of Piezo2-containing proprioceptive terminals could be the cause of the aforementioned primary damage in DOMS [3,4]. Moreover, the proposed 1–2 day time window of an acute Piezo2 channelopathy overlaps the complete return of cardiac parasympathetic activity to pre-exercise levels after high-intensity exercise, indicating an imbalanced autonomic control [3].

The orthostatic stress test (OST) is a widely used measure to evaluate cardiac autonomic responsiveness in cardiology [11]. Hence, our goal was to examine any indication of decreased orthostatic tolerance as a result of unaccustomed fatiguing exercise with a successive isometric weight-loaded wall-sit test, as well as to find any distinguishable alterations due to the DOMS effect. Our aim for this investigation is especially interesting in light of a recent finding that static isometric exercise, like a wall-sit, best serves resting blood pressure in the long run, in contrast to other exercise methods, like dynamic resistance training, high-intensity interval training, etc. [12]. The theorized peripheral intrafusal Piezo2 channelopathy-induced transient disruption of Piezo1–Piezo2 crosstalk and NMDA activation in DOMS could be the reason why orthostasis may be impaired. 

Another suspicion was that DOMS could abnormally alter diastolic blood pressure (BP) and heart rate (HR) during the OST after DOMS-inducing fatiguing exercise. The ground for this suspicion was that diabetes mellitus can cause a neuron terminal microinjury similar to the mechano-energetic one proposed in DOMS. This analogy may be used regardless of profound differences between these insults [13]. Correspondingly, abnormal diastolic BP and HR are a consequence of tilting in diabetes mellitus, indicating an impaired autonomous nervous system [14]. Furthermore, recent findings showed atypical hippocampal-like metabotropic PLD-mGluR on intrafusal primary afferents that were homomeric to metabotropic GluK2 [15]. Of note is that the GluK2 receptor has a role in glucose homeostasis maintenance [16]. Additionally, mGluRs contributes to glucose-stimulated insulin secretion in pancreatic beta cells [17], and Piezo1 has a critical role in this process [18]. That being so, a further aim was to specifically test diastolic BP and HR alterations in DOMS during the OST.

## 2. Materials and Methods

### 2.1. Study Design

We performed a prospective observational study with the participation of the Hungarian Junior Swim Team in March 2022. Informed consent was obtained from all subjects and/or their legal guardian(s). The Semmelweis University Regional and Institutional Committee of Science and Research Ethics (SE RKEB 282/2021) and the Hungarian Central Ethics Committee (ETT TUKEB IV/10282-1/2020/EKU) approved the study. 

### 2.2. Participants

Altogether, 19 junior swimmers were included in our study. All participants had qualified for the European Championships in summer 2022. As all of the athletes were preparing for the same competition that year, they had the same training status during our examinations. The athletes were asked to carry out their usual routine on the day of the test, which included eating breakfast and consuming an adequate amount of fluids. After the execution of the exercise protocol of the current study, 16 athletes reported DOMS (Table 1). 

### 2.3. Procedures

**Figure 1:**
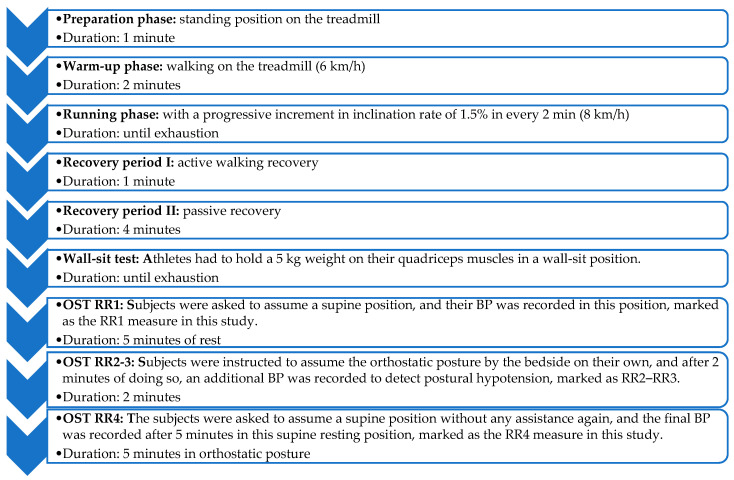
Flowchart of the exercise protocol of the current pilot study followed by an orthostatic stress test (OST).

The sports cardiology screening protocol and weight-loaded wall-sit test were the same as described in our earlier study [19], and an OST followed (Figure 1).

#### 2.3.1. Sports Cardiology Screening with Wall-Sit Test

The detailed sports cardiology screening contained a sports-specific questionnaire, laboratory test, resting electrocardiogram (ECG), echocardiography, body composition analysis, and cardiopulmonary exercise test (CPET). All of the examinations were performed by the same medical staff.

The sports-specific questionnaire assessed the athletes’ personal medical history. We particularly focused on the muscle soreness level of the athletes, which was assessed before the screening, 24 h after the screening, and 48 h after the screening on a 1 to 10 visual analog scale (VAS).

We used the same exercise protocol as described in our earlier study [19]. It contained a 1 min preparation phase in a standing position, a 2 min walking warm-up phase (6 km/h), a running phase (8 km/h) with a progressive increment in inclination rate of 1.5% every 2 min until exhaustion, and a recovery period with 1 min active walking recovery and 4 min passive recovery. The athletes were instructed not to hold the handrail. Of note is that swimmers are unaccustomed to this running test, as the repeated loading of the anti-gravitational muscles in a gravitational force environment significantly differs from the load on these athletes’ musculoskeletal system in the water. Therefore, the occurrence of DOMS in the case of some swimmers in the study sample could be expected even from this part of the exercise protocol.

The weight loading of the lower extremities was carried out after the running test using a wall-sit test. During the wall-sit test, the athletes had to hold a 5 kg weight on their quadriceps muscles in a wall-sit position until exhaustion. Important to note, again, is that not only are swimmers unaccustomed to the isometric weight-loaded wall-sit but also that it could induce DOMS.

Resting ECG was performed with a system called CardioSoft PC (GE Healthcare, Helsinki, Finland). Laboratory diagnostics contained a qualitative and quantitative complete blood count, creatine kinase, creatine kinase-MB, highly sensitive troponin T, and NK cell activity. A blood test was carried out two times, before and after the exercise and wall-sit tests, on venous blood samples. A GE T-2100 treadmill ergometer was used to detect cardiorespiratory fitness (Healthcare, Finland). Gas parameters were calculated with a breath-by-breath automated cardiopulmonary exercise system (Respiratory Ergostik, Geratherm, Bad Kissingen, Germany). The athletes were encouraged to reach maximal exertion, which was confirmed by the respiratory exchange ratio and by reaching the predicted maximal HR and VO_2_ values.

#### 2.3.2. Orthostatic Stress Test

BP was measured with an oscillometric method-based validated automatic electric device (Omron Healthcare Co., Tokyo, Japan) on the left arm. The subjects were asked to assume a supine position with 5 min of rest, and their BP was recorded in this position, marked as the RR1 measure in this study. Afterwards, the subjects were instructed to assume the orthostatic posture by the bedside on their own, and after 2 min of doing so, an additional BP was recorded to detect postural hypotension, marked as the RR2 and RR3 measures in this study. After 5 min in the orthostatic posture, the subjects were asked to assume a supine position without any assistance again, and the final BP was recorded after 5 min in this supine resting position, marked as the RR4 measure in this study.

### 2.4. Statistical Tests

Variables for the selected samples are presented as means and standard deviations. For the purpose of selecting an adequate statistical procedure and considering the limited sample size, Shapiro–Wilk’s W test of normality was carried out. For the comparison of the measured datasets, an independent sample t-test and the Mann–Whitney U test were performed. Statistical calculations were executed with Statistica version 12 software (StatSoft Europe GmbH, Hamburg, Germany) and JASP software (jasp-stats.org, installed version 0.16). The significance level was determined in all cases at *p* < 0.05.

## 3. Results

### 3.1. Orthostatic Hypotension

The findings show that every single subject, regardless of the DOMS effect, who undertook the OST experienced orthostatic hypotension (OH) after the exercise protocol, according to the guidelines of the European Society of Cardiology (ESC). Correspondingly, a systolic BP fall of ≥20 mmHg from a baseline value, a diastolic BP fall of ≥10 mmHg, or a systolic BP decrease to <90 mmHg was seen among the investigated athletes (Table 2 and Table 3).

### 3.2. VAS Survey

Based on the VAS surveys, the muscle soreness level of the athletes was assessed before the screening, 24 h after the screening, and 48 h after the screening. Only 3 out of 19 athletes did not report DOMS (Table 1).

### 3.3. Decreased Diastolic BP in DOMS

The current study also discovered that diastolic BP was significantly decreased in the DOMS group, where the mean absolute difference of diastolic BP was 9.68 mmHg (SD: +/−5.38) in the standing position after the supine position during the OST, in contrast to the group that did not experience DOMS, where the mean absolute difference of diastolic BP was only 2.22 mmHg (SD: +/−1.38). To quantify dysregulation differences in BP from RR1 to RR2, the individual values were calculated and the average and SD values were determined for the groups with and without DOMS.

### 3.4. Dysregulated HR in DOMS

Significant dysregulation of HR was observed in the group of athletes who experienced DOMS when they assumed a supine position after the sustained standing position during the OST, in contrast to the group of subjects who did not experience DOMS. To quantify dysregulation differences of individual values from group means, absolute differences from the group means were calculated for the participants’ data values individually, and average (called “statistical variability” in Table 4) and SD values were determined from these derived values for the groups with and without DOMS.

### 3.5. Substantial Drop in HR in Sustained Standing Position in Some DOMS Subjects

Interestingly, the sustained standing position substantially decreased HR below the HR of the supine position in only three subjects; hence, HR3 was substantially lower than HR1 (Table 5). All three subjects experienced a DOMS effect afterwards. This drop in HR came after HR increased in basically every single subject when they stood up from the supine position; hence, HR2 was higher than HR1.

### 3.6. Self-Reported Dizziness

Six participants experienced self-reported dizziness during the standing position, and interestingly, all subjects were from the group that experienced DOMS.

## 4. Discussion

The primary aim of the current pilot study was to use an OST to test whether orthostatic tolerance is reduced due an acute intensive exercise protocol combined with a wall-sit isometric one. The secondary aim was to use an OST to test whether we could see any distinguishable alteration in diastolic BP and HR due to the DOMS effect, as is observed in diabetes. Indeed, our study demonstrated reduced orthostatic tolerance in our subjects and found identifiable alterations due to the DOMS effect, resembling the ones experienced in diabetes.

The OST is a widely adopted measure to evaluate cardiac autonomic responsiveness in cardiology [11]. The guidelines of the ESC changed the definition of OH in 2018. Consequently, it refers to a fall in systolic BP from a baseline value of ≥20 mmHg, a fall in diastolic BP of ≥10 mmHg, or a decrease in systolic BP to <90 mmHg [20]. In our study, all subjects fell under these defined categories after they executed our exercise protocol followed by an OST regardless of whether they had experienced DOMS. However, our investigation showed a further differentiated indication of autonomic dysregulation in subjects who had experienced DOMS.

It has long been known that regular exercise training increases plasma and blood volume [21]. The positive correlation between plasma and blood volume with orthostatic tolerance has also been noted, as well as the negative correlation between baroreceptor sensitivity and orthostatic tolerance [22]. Moreover, it was demonstrated that an enhancement in orthostatic tolerance can be accomplished through an increase in plasma volume and a decrease in baroreceptor sensitivity in untrained subjects [23]. Furthermore, in intensive acute exercise (IAE), increased hemoconcentration can elevate shear stress detection through endothelial Piezo1 in Piezo2 of somatosensory terminals in resistance arteries. As a result, Piezo1 in smooth muscle cells of resistance arteries can also contribute to vascular dilatation. Additionally, Piezo1 can regulate osmolality and water outflow dynamics [24,25], possibly in correlation with exercise intensity and especially in fatigue-induced somatosensory hyperexcitability [26]. The intimate excessive cross-activation and hyperexcitation of Piezo1 and Piezo2 ion channels, together forming the Piezo system [27], could be one good explanation for the decreased orthostatic tolerance as a consequence of fatiguing IAE.

Nonetheless, the regulation of orthostatic challenge does not stop here if we take a neurocentric view. When fatigue restrains muscle performance and force production cannot be sustained or enhanced cognitively, then an overreaching response can be induced. This response is often used by coaches in training sessions [6,28,29,30]. This overreaching response, or the extension of homeostasis, is also called “allostasis” when it comes to neuroenergetics, meaning the sustainment of stability in a perturbed environment when the neural energy demand is high [31], as could be the case in IAE. Repeated overreaching training sessions with intermittent recovery periods could result in an elevated level of homeostasis. This adaptation, with an increased homeostasis level, is called “supercompensation” [6,30,32] and is guided by the autonomic nervous system, like in the case of the overreaching response [29]. Both processes involve motor learning and memory through the use of the proprioceptive system [6,30]. The homeostatic driver of the overreaching response is an acute stress response (ASR) [6,30]. The proposed critical gateway in this allostatic stress time window are the Piezo2 channels at the proprioceptive sensory terminals [27]. The inactivation of Piezo2 channels in response to hyperexcitation during allostatic stress is a homeostatic response [3,33]. Correspondingly, a recent theory postulated that Piezo2 ion channels can be inactivated in baroreceptors during IAE [34]. In that case, the Ca_v_1.3 channels in the sinoatrial node (SAN) could be unopposed, with ever-increasing pacemaking during the sustained IAE activity. Hence, it means that Piezo2 inactivation in baroreceptors could be the excitatory neural upper limit of entrainment activity between baroreceptors and SAN [34]. However, it is suggested that synchronization deactivation is not complete because Piezo1 can maintain residual entrainment activity from this aspect [34]. The current authors interpret that the athletes produced the observed positive OST after the exercise protocol due to the insufficient abrupt return of Piezo2 reactivation-induced entrainment between baroreceptors and SAN, likely accompanied by hypovolemia and transiently increased hemoconcentration, in which mechanism Piezo1 proteins are also suggested to have a primary role.

Nevertheless, the overreaching response could go beyond homeostatic limits, and that is the proposed primary damage or transient Piezo2 channelopathy [4]. It was noted earlier that the return of cardiac parasympathetic activity returns to pre-exercise levels only 1 to 2 days after high-intensity exercise [29], which is an overlap with the time window of the proposed transient Piezo2 channelopathy in DOMS [3]. Hence, functional crosstalk is suspected between the autonomic nervous system and the proprioceptive Piezo2 ion channels [3,35]. Correspondingly, it was put forward that Piezo2 channelopathy-derived subthreshold-imbalanced Ca^2+^ currents could induce NMDA activation, and the activated NMDA receptors could have a central signaling role in this functional crosstalk during the aforementioned transient autonomic disbalance, not to mention that Piezo1–Piezo2 crosstalk might have additional importance in this process [4]. It was argued that transient Piezo2 channelopathy also means a transient loss of crosstalk between Piezo1 and Piezo2 [27]. This impaired crosstalk could have significant relevance, because Piezo1 channels have the features to increase the performance of the whole body by resetting cardiovascular homeostasis [36] beyond sensing and responding in a spatially restricted manner [37]. As a result of Piezo2 deactivation in baroreceptors, the residual Piezo1 activity could be disturbed in the presence of the transient loss of Piezo1–Piezo2 crosstalk elsewhere. The authors of this paper translate this phenomenon as the DOMS-induced lost Piezo1–Piezo2 crosstalk promoting NMDA activation, which could impair diastolic depolarization and hence could induce the observed decrease in diastolic pressure in DOMS as a consequence of post-exercise OST. Indeed, activated NMDA receptors can increase the pacing frequency in a single SAN pacemaker cell, and these pacemaker cells are demonstrated to have glutamatergic neuron-like features [38]. This influence of increased NMDA activation could possibly impair the pacemaker potential or the slow diastolic depolarization in the absence of Piezo1–Piezo2 crosstalk. Ca_v_1.3 L-type calcium channels have a central role in the initiation of this rapid depolarization, and, not surprisingly, Ca_v_1.3 knockout mice cannot maintain stable pacing, experiencing bradycardia and intermittent sinus arrests [39,40]. Interesting to note is that, in proprioceptive signaling, when Piezo2 is inactivated, then Na_v_1.1 takes over the encoding [41]. As an analogy, the role of sodium channels in SAN is not entirely known; however, the mutation of the cardiac-specific voltage-gated sodium channel Na_v_1.5 isoform-encoding SCN5A gene in SAN causes cardiac conduction disease [42]. The current authors propose that, when Piezo2 is activated in baroreceptors and synchronized with the cardiac vagal afferents, then cardiac-specific Na_v_1.5, in collaboration with Ca_v_1.2 and Ca_v_1.3, maintains the proper pacing and conduction in the SAN. On the contrary, when Piezo2 is deactivated in baroreceptors, entrainment is suggested to diminish to undetectable levels, and Ca_v_1.3 could gain dominance in pacemaking [34,43]. Indeed, animal research shows that Ca_v_1.3 is needed for beta-adrenergic-triggered automaticity in SAN pacemaker cells [44]. However, when transient, chronic, or terminal Piezo2 microinjury is present in the Piezo system, the dominance of Ca_v_1.3 is proposed to be sustained without proper neural plasticity [45], and possibly in pacemaking as well. Therefore, activated NMDA receptors could impair slow diastolic depolarization, leading to an increased reduction in diastolic BP in this perturbed state, as was observed in the athletes with DOMS during the post-exercise OST.

Of note is that the primary damage in DOMS is proposed to be a Piezo2 channelopathy with associated impairment of glutamate vesicular release [5]. Hence, it is theorized that the glutamate vesicular release of SAN cells with glutamatergic features could be also impaired, leading to increased activation of NMDA receptors in the central nervous system. Moreover, the increased glycolytic metabolism and excessive excitation of activated voltage-gated sodium channels in these types of neurons could increase extracellular acidification [46], possibly even in SAN pacemaker cells. This proposed mechanism could be analogous with the impaired lactate shuttle mechanism postulated in DOMS, which certainly implies perturbed glucose metabolism [47]. Furthermore, this extracellular acidification process could activate cardiac acid-sensing ion channel 3 (ASIC3) exclusively on sensory neurons, like in myocardial ischemia [48] and as suggested in DOMS [47].

After all, Piezo2 channelopathy and impaired glutamate vesicular release in DOMS could have relevance in cardiac pacemaking, in conjunction with activated NMDA receptors and activated ASIC3 ion channels [45], having led to the observed significant diastolic BP drop and impaired HR adaptation as a consequence of the OST after unaccustomed fatiguing IAE with a successive weight-loaded isometric wall-sit.

It is a common agreement among scientists that impaired proprioception is associated with higher risk of sport injuries. The current finding that DOMS not only reduces proprioception but also impairs orthostasis when it is induced by unaccustomed fatiguing intensive acute concentric exercise with a successive isometric weight-loaded wall-sit could additionally explain why DOMS may contribute to higher injury incidence.

The low number of athletes without DOMS in the current pilot study is recognized; however, this limitation could not have been predicted or controlled in advance. A possible source of bias could have been the homogenous population sample, and the top athletes may represent only a minority of the general population. Accordingly, in our view, it is important to reflect upon these observations with a novel mechanistic interpretation in reference to the differences between the group with DOMS and the group without DOMS in spite of the fact that the sample size was low for the group without DOMS.

Finally, further research is needed to investigate the function of Piezo2 channels, which are not only responsible primarily for proprioception but also for constructing the molecular transduction mechanism of baroreception. Future studies are proposed to be conducted on larger sample sizes to investigate the observed phenomena in DOMS, along with an examination of the time window of the observed impaired orthostasis, especially considering that the proposed acute Piezo2 channelopathy could last 1–2 days, which leads to a complete return of cardiac parasympathetic activity to pre-exercise levels after high-intensity exercise.

## Figures and Tables

**Table 1:** Baseline characteristics (mean +/− SD) of the sample (VAS: visual analog scale, VO_2_ max: maximal aerobic capacity).

	With DOMS (*n* = 16)	Without DOMS (*n* = 3)
Age (years)	16.8 ± 2.9	15.3 ± 2.1
Female (*n*,%)	8 (50%)	2 (67%)
Training (hours/week)	21.1 ± 2.2	18.7 ± 2.3
Training history (years)	11.9 ± 3.5	10.3 ± 1.2
Last training before exam (h)	28.5 ± 18.2	13.7 ± 8.5
Last training length (min)	103.8 ± 39.8	110.0 ± 45.8
Wall-sit time (s)	119.2 ± 44.0	169.4 ± 39.0
Muscle fever pre-exercise (VAS ^1^ 1–10)	1.7 ± 2.1	-
Muscle fever post-exercise 1st day (VAS ^1^ 1–10)	3.8 ± 2.3	-
Muscle fever post-exercise 2nd day (VAS ^1^ 1–10)	3.5 ± 2.3	-
Load time (min)	13.4 ± 1.0	13.2 ± 1.6
Peak lactate (mmol/L)	10.1 ± 2.7	11.6 ± 1.4
Restitution lactate (mmol/L)	9.6 ± 2.9	9.4 ± 1.9
VO_2_ max ^2^ male (mL/kg/min)	58.0 ± 1.8	59.6 ± 0
VO_2_ max ^2^ female (mL/kg/min)	52.9 ± 3.1	50.1 ± 2.5
VO_2_ max ^2^ (mL/kg/min)	55.5 ± 3.6	53.3 ± 5.8
Volume consumption (mL)	500.0 ± 316.2	733.3 ± 251.7

^1^ VAS: visual analog scale, ^2^ VO_2_ max: maximal aerobic capacity.

**Table 2:** Responses in the systolic BP values (mean +/− SD) during the different measurement points (RR1 to RR4) in the orthostatic stress test. Significant difference in systolic BP data between RR1–RR2 (^a^) and RR3–RR4 (^b^) (*p* < 0.05).

	RR1 ^1^	RR2 ^2^	RR3 ^3^	RR4 ^4^
Systolic BP (mmHg) average	156.1 ^a^	111.2 ^a^	113.1 ^b^	129.9 ^b^
SD	14.6	22.1	17.6	13.3

^1^ RR1: The subjects were asked to assume a supine position after the exercise protocol, and their BP was recorded in this position, marked as the RR1 measure in the study. ^2^ RR2: After, the subjects were instructed to assume the orthostatic posture by the bedside on their own, and BP was recorded, marked as the RR2 measure. ^3^ RR3: After 2 min of doing so, an additional BP was recorded to detect postural hypotension, marked as RR3. ^4^ RR4: Finally, the subjects were asked to assume a supine position without any assistance again, and the final BP was recorded after 5 min in this supine resting position, marked as the RR4 measure in this study.

**Table 3:** Average and SD diastolic BP values of every subject for the RR1 to RR4 measurements.

	RR1	RR2	RR3	RR4
Diastolic BP (mmHg) average	80.7	75.8	72.1	73.6
SD	9.7	18.1	12.4	11.2

**Table 4:** Average and SD of absolute difference from group means (statistical variability) for HR3 and HR4 for the groups with and without DOMS. The values indicate significant difference between the two groups (^a^,^b^) (*p* < 0.05).

HR Absolute Statistical Variability				
	With DOMS		Without DOMS	
	HR3 ^1^	HR4 ^2^	HR3 ^1^	HR4 ^2^
Average	16.4 ^a^	12.7 ^b^	2.6 ^a^	0.4 ^b^
SD	11.2	21.1	1.5	0.2

^1^ HR3: After 2 min of the orthostatic posture, an additional heart rate (HR) was recorded, marked as the HR3 measure. ^2^ HR4: Finally, the subjects were asked to assume a supine position without any assistance again, and the final HR was recorded after 5 min in this supine resting position, marked as the HR4 measure in this study.

**Table 5:** Three subjects experiencing DOMS, with substantially lower HR3 than HR1.

Subjects Experiencing DOMS, with Substantially Lower HR3 than HR1		
	HR1 ^1^	HR3 ^2^
Subject 1	143	74
Subject 2	109	74
Subject 3	119	79

^1^ HR1: Subjects were asked to assume a supine position after the exercise protocol, and their heart rate (HR) was recorded in this position, marked as the HR1 measure in this study. ^2^ HR3: The subjects were asked to assume a supine position without any assistance again, and the third HR was recorded after 5 min in this supine resting position, marked as the HR3 measure.

## Data Availability

The data presented in this study are available on request from the corresponding author.
